# Predicting Engagement With Conversational Agents in Mental Health Therapy by Examining the Role of Epistemic Trust, Personality, and Fear of Intimacy: Cross-Sectional Web-Based Survey Study

**DOI:** 10.2196/70698

**Published:** 2025-07-30

**Authors:** Fanny Guglielmucci, Daniela Di Basilio

**Affiliations:** 1 Department of Philosophy, Communication and Performing Arts, Roma Tre University, Rome, Italy; 2 Faculty of Health and Medicine, Division of Health Research, Lancaster University, Sir John Fisher Drive, Lancaster, LA1 4AT, United Kingdom, 44 07312255301

**Keywords:** artificial intelligence, AI, conversational agents, CA, mental health, psychotherapy, epistemic trust, personality

## Abstract

**Background:**

The use of conversational agents (CAs) in mental health therapy is gaining traction due to their accessibility, anonymity, and nonjudgmental nature. However, understanding the psychological factors driving preferences for CA-based therapy remains critical to ensure ethical and effective application. Variables such as epistemic trust, attachment styles, personality traits, and fear of intimacy appear central in shaping attitudes toward these artificial intelligence (AI)–driven interventions.

**Objective:**

This study aimed to investigate the role of epistemic trust, attachment styles, personality traits, and fear of intimacy in influencing individuals’ willingness to engage with CA-based therapy.

**Methods:**

An online survey was administered to 876 psychology students, yielding 736 responses (84.01% response rate). Variables measured included epistemic trust, attachment styles, personality traits, and fear of intimacy. A 5-point ordinal scale assessed willingness to engage in CA-based therapy. The data were analyzed using ordinal logistic regression models, including proportional odds models (POMs), nonproportional odds models (NPOMs), and partial proportional odds models (PPOMs), with residual deviance used to compare model fit.

**Results:**

The PPOM provided the best model fit (residual deviance=3530.47), outperforming both the NPOM (deviance=6244.01) and the POM based on Brant test results indicating violations of the proportional odds assumption (*χ*²_105_=187.8; *P*<.001). In the final model (n=735), epistemic trust significantly increased willingness to engage in CA-based therapy across all ordinal thresholds (odds ratio [OR] 1.75, 95% CI 1.50, 2.03; *P*<.001). Fear of sharing demonstrated a nonuniform effect, with stronger associations at higher levels of willingness (OR 1.086; *P*=.001). Among personality traits, detachment negatively predicted CA preference (OR 0.95; *P*=.001), while psychoticism showed a positive association (OR 1.12; *P*=.003). Being single emerged as a strong predictor of preference for CA-based therapy (OR 3.717; *P*<.001). Attachment styles showed more nuanced effects. While dismissing and fearful-avoidant individuals were descriptively less inclined to engage in traditional human-based therapy, this association was nonsignificant in the case of fearful-avoidant attachment (*P*=.34) and should therefore be interpreted cautiously.

**Conclusions:**

Epistemic trust and fear of intimacy emerged as pivotal factors influencing preferences for CA-based therapy, underscoring the role of interpersonal dynamics and emotional vulnerabilities. The findings suggest that individuals with avoidant attachment styles or maladaptive personality traits are more inclined toward AI-mediated interventions, driven by reduced fear of judgment and increased perceived safety. The relative homogeneity of the sample considered—particularly in terms of age, education level, and cultural exposure—limits the generalizability of these findings to broader or more diverse populations. Nonetheless, these insights highlight the need for ethical considerations and personalized approaches in deploying CA-based mental health tools to balance user reliance with human-centric therapeutic values.

## Introduction

### Background

In the past few years, the way clinical professionals, patients, and the general population relate to emotional suffering has notably changed. A crucial role in this shift can be attributed to the rise of artificial intelligence (AI) and conversational agents (CAs). These are advanced manifestations of large language models, which are capable of analyzing and extracting knowledge from human conversations, using it to adaptively prompt a real-time answer and consequently creating multiturn human-computer dialogues. The unique ability of CAs to interact with human beings, understand their communication styles, and engage in human-like conversational responses gained notable attention. This is due primarily to their potential applications in the fields of enhancing mental well-being and providing social support [1], with CA-based tools now assuming more nuanced roles, serving not only as companions [2] but also as therapists for individuals seeking emotional support and guidance (for reviews and meta-analyses, see, for example, [3-6]). The rising trend of using CAs for improving mental health can be attributed to various factors. First, the widespread integration of AI-driven technologies in daily life has normalized human-like interactions with machines. CAs, designed with advanced natural language processing capabilities, can engage users in conversations that mimic human interaction, thereby potentially helping alleviate feelings of loneliness [2,7,8]. In addition, their anonymity and nonjudgmental nature may appeal to individuals who might feel hesitant or stigmatized about sharing their emotions with their human counterparts [1,9,10].

However, beyond these technical features, it is imperative to understand the psychological determinants that may drive individuals to rely solely, or primarily, on CA-based therapy, bypassing human interaction. This understanding is crucial for the ethical provision of mental health care, as for some (eg, people with a poor mental capacity or severe mental illness), the prevalent use of CAs may be counterproductive, for example, by eliciting dependence and overreliance on the relationship built with the CA [11,12].

Several psychological factors may influence a person’s willingness to communicate exclusively with autonomous agents in order to deal with life-related painful emotions and memories. Among those, 4 factors appear to be of major importance, as pointed out by several reviews on this topic: epistemic trust, fear of intimacy, attachment strategies, and personality domains (for more details on these reviews and meta-analyses, see sections below).

### Epistemic Trust in AI

Perceptions of AI capabilities and trust in its effectiveness may play a significant role in shaping individuals’ attitudes toward CA-based therapy. Over the past decade, there has been an incremental growth in the number of reviews [13-16] and meta-analyses [17,18] on the factors influencing trust in AI. Riedl [14] suggested that trust in technology relies on “trust propensity,” that is, the tendency to believe that the use of AI can be beneficial and that its performance will appropriately and consistently allow it to address specific tasks. However, such beliefs are not necessarily accurate, concealing a logical fallacy deeply rooted in technology idealization [19]. In the case of CA-based therapy, for example, people may overlook the possibility that relying on AI-driven support alone may compromise their capability to think critically and process information autonomously. In addition, they might be exposed to the risk of receiving information that might not be fully accurate and reliable—or might, in some cases, even be dangerous [20,21].

As Alvarado [22] pointed out, AI is primarily an “epistemic technology,” designed to enhance human knowledge in different epistemic contexts (eg, inquiry and accurate prediction). To be used epistemologically, though, AI needs to “earn” our epistemic trust, that is, the tendency to believe in shared communicated information and consider the source of information reliable and trustworthy [23].

As a concept, epistemic trust has a long history that precedes the rise of AI. For instance, Fonagy et al [24] have proposed conceptualizing epistemic trust within a broader sociocommunicative perspective as the output of (early) social learning processes. In these processes, several contextual factors (ie, attachment and personality styles, mentalizing capabilities, peers, and sociocultural environment) interact with each other, influencing the way we gradually discern who is knowledgeable and trustworthy [24-26]. In today’s world, AI and social media seem to play a pivotal role [27] in becoming a primary source of our knowledge. However, our ability to trust what others say— including CAs—relies on the extent to which we feel safe in our relationships with them [28]. Indeed, individual differences in the propensity to place trust in and engage with AI are underpinned by various subjective factors, including, for example, personality traits and characteristics [29,30], which could help predict the likelihood of individuals opting for AI-based therapy over human-based clinical interventions.

In this study, epistemic trust was conceptualized as the individual’s predisposition to perceive the information delivered by a CA as accurate, relevant, generalizable, and safe, and therefore, the CA as a trustworthy communicator. This is in line with different definitions of epistemic trust (eg, [23,31]; reflecting both the tendency to believe in shared communicated information and to consider the source of that information as reliable and trustworthy). In doing this, this study drew from the established use of this concept in clinical psychology, where epistemic trust encompasses both the informational content and the relational qualities of the communicator. In therapeutic contexts, the trust one places in the message and in the person delivering it is widely considered inseparable, as effective psychological interventions rely on both epistemic validity and emotional resonance. Accordingly, our study treats epistemic trust as a relationally embedded construct, capturing both the individual’s openness to the information provided by a CA and their implicit trust in the agent as a reliable, nonthreatening source.

### Attachment-Based Personality Development and Propensity to Trust AI

Research efforts aimed at understanding attitudes toward AI have primarily concentrated on personality traits underpinning our preferences and behaviors [32,33], leading to the identification of 2 main theoretical conceptualizations of personality and trust in AI. The first considers personality traits as predictors of trust, while the second sees trust as a mediator between personality and behavioral intentions toward AI systems (for a systematic review of the research status on personality and trust in the context of AI systems, see Riedl [14]). In this regard, the same author [14] proposed a third framework suggesting that the propensity to trust AI is a specific personality trait related to broader personality constructs (ie, the Big Five). This framework also suggests a complex role for emotions and affective states, which are influenced by personality traits and, in turn, influence the development of trust in others and how we relate to them [34].

Similarly, Fonagy and colleagues [35] recently affirmed that “attachment and personality styles are seen as communicative strategies underpinning social learning to ensure adaptation to ever-changing social situations.” In other words, deeply rooted aspects of individuals’ selves, such as their personality patterns and attachment styles, influence their emotional regulation skills and abilities to relate to others. The quality of our interactions with others, in turn, shapes our inner generalized perception of the availability and trustworthiness of our other people and the overall environment [27,36].

Although research in this field is still in its infancy, recent findings [28] suggest that attachment insecurity can predict a lack of trust in AI. In addition, the same study [28] found that exposure to attachment security cues (but not positive affect) via priming or nudging increased trust levels. In essence, these findings are closely akin to Fonagy’s conceptualizations of epistemic trust, personality, attachment, and mentalizing skills, suggesting a possible predictive role of epistemic trust, personality, and attachment in our disposition toward CA-based therapy.

### Fear of Intimacy and Avoidance-Based Attachment Patterns

Intimacy involves the capacity to commit oneself to particular affiliations and relationships, and to possess the “ethical courage” necessary to uphold these commitments [37]. Intimate relationships develop in early infant-caregiver relationships, fostering trust in others and instilling the confidence and self-esteem necessary for a child to explore their social environment [38,39]. Difficulties in building intimate relationships during adolescence and early adulthood reflect a lack of emotional integration and have been linked with a wide variety of mental health problems, including general maladjustment and personality disorders [40,41].

Currently, technology often plays a paradoxical role in fostering a sense of “virtual intimacy” while leading to potential social isolation [42,43]. This dichotomy may be underpinned by the coexistence of a desire for connection with a fear of face-to-face interactions, where intimacy and empathy are truly tested [44,45]. This dynamic may also be found in therapeutic relationships, where patients may desire, albeit feel uncomfortable, to open up about their personal lives to mental health professionals [46]. From this perspective, the desire to interact solely with AI to address psychological distress may mask a deep fear of intimacy while simultaneously reflecting the need to understand and make sense of our emotionally painful experiences.

Individuals grappling with the fear of intimacy typically exhibit characteristics rooted in 2 distinct avoidance-based attachment strategies, which correspond to 2 different models of the self: fearful and dismissing [47]. In both cases, others are perceived as untrustworthy and threatening our security. However, individuals with a fearful attachment style consciously desire social connection despite avoiding them due to a depleted sense of self-worth and a deep-seated fear of rejection. They tend not to believe they deserve love and support and mistrust others’ intentions. Conversely, subjects who dismiss intimacy possess a positive self-representation and defensively deny their need or desire for intimate social contact. This defensive dismissal serves to protect their self-image and independence [47-49]. This differentiation suggests that both groups may feel more secure in an AI-based relationship and be more prone to trust CAs rather than human beings (eg, therapists), albeit for different reasons: the former (fearfully attached) because of the fear of opening up to someone who might hurt, judge, or reject them, while the latter (avoidantly attached) as they value connections that allow them to preserve their positive self-image and independence [50-52].

In the past few years, an increasing reliance on CAs in mental health therapy has emerged [1], driven by their advanced ability to simulate human-like interactions and their appeal as anonymous, nonjudgmental support systems. While the technical capabilities of CAs have been extensively explored, less is understood about the psychological factors influencing individuals’ preference for CA-based therapy over human interaction. Key constructs such as epistemic trust, attachment strategies, personality traits, and fear of intimacy have been identified as critical determinants of this preference.

Considering this, this study aimed to address a gap in the literature by examining how key psychological factors—specifically epistemic trust, personality traits, avoidance-based attachment styles, and fear of intimacy—influenced individuals’ willingness to engage with CAs for mental health support. The goal was to contribute to a deeper understanding of the psychological underpinnings that shape preferences for CA-based therapy, with broader implications for the ethical implementation of AI in mental health care. To achieve this, we employed an ordinal logistic regression model to assess the predictive role of these factors in shaping individuals’ dispositions toward AI-mediated therapeutic interventions.

## Methods

### Recruitment and Sample

Data were collected through an online survey distributed to students attending the MSc course “Psychology of Communication” at the Department of Philosophy, Communication and Performing Arts of Rome3 University (Italy). The study was advertised by posting an e-copy of the study flyer on the academic page and Microsoft Teams channel of the MSc course, reaching out to a pool of 876 students. Participants accessed the survey through a Google Forms link, where they were presented with a Participation Information Sheet (PIS) outlining study details, that is, a concise overview of the study’s objectives, participation requirements, data handling procedures, and contact information for the principal investigator, followed by a consent form.

### Ethical Considerations

The participants were asked to confirm their agreement to participate by clicking “I agree” on the consent form. Selecting this option to express informed consent redirected participants to another form containing the assessment measures used as part of this study. Participants remained anonymous and could withdraw from the study at any time before data analysis by contacting the principal investigator through email as described in the PIS. The online survey used in this study was designed and implemented in accordance with the CHERRIES (Checklist for Reporting Results of Internet E-Surveys [53]) guidelines, ensuring methodological transparency, ethical compliance, data integrity, and robust participant engagement throughout the process. Table 1 reports the CHERRIES criteria and how they were met for this specific study. All procedures performed in studies involving human participants were in accordance with the ethical standards of Italian Psychoanalytical Association (ethical approval 0405/2023) and with the Declaration of Helsinki of 1975, revised in 2013.

**Table 1.** CHERRIES^a^ compliance.

CHERRIES criteria	Study implementation details
Design	Cross-sectional web survey design using Google Forms.
IRB^b^ approval and informed consent process	IRB approval obtained (AIPsi^c^ ethical approval 0405/2023); informed consent collected through an online form.
Development and pretesting	The survey was pretested internally by the research team; translated scales were validated via back-translation.
Recruitment process and description of the sample having access to the questionnaire	Participants were MSc students recruited through an e-flyer advertising the study, posted on the MSc course Microsoft Teams channel and University webpage; 876 were invited.
Survey administration	Google Forms used; PIS^d^ and consent form provided before questionnaire; anonymity and withdrawal rights ensured.
Response rate	736 out of 876 responded (84.01% response rate); 735 included in final analysis after removing 1 outlier.
Preventing multiple entries from the same individual	Participants could only submit the form once; the survey required institutional login preventing duplicate submissions.
Analysis	Ordinal logistic regression models used; assumptions tested; best-fit model selected (PPOM^e^); power analysis conducted.

a CHERRIES: Checklist for Reporting Results of Internet E-Surveys.

b IRB: institutional review board.

c AIPsi: Italian Psychoanalytical Association.

d PIS: Participation Information Sheet.

e PPOM: partial proportional odds model.

### Measures

#### Sociodemographic Information and Willingness to Rely on CA-Based Therapy

Sociodemographic information (eg, age, gender, years of education, and marital status) was collected. To assess the preference for CA-based therapy over human being-based clinical interventions, participants were asked to answer the following question: “On a scale from 1 to 5, how willing are you to participate in psychotherapy sessions solely facilitated by artificial intelligence (AI), without any interaction with a human psychologist?” (scores range from 1=not at all to 5=extremely).

#### Epistemic Trust

The Italian adaptation [54] of the Epistemic Trust, Credulity and Mistrust Questionnaire (ETCMQ [31]) was used to assess epistemic trust. This is a 15-item self-report measure with a 3-factor structure assessing various forms of epistemic stance (ie, epistemic trust, credulity, and mistrust). Items were adapted and referred specifically to CA-based therapy (ie, When I have a personal problem, I usually ask AI for advice; If I don’t know what to do, my first impulse is to ask AI because I trust). In this study, Cronbach α for ETCMQ was 0.95.

#### Attachment

The Italian translation of the Relationship Questionnaire [55] (RQ [47]) was used to assess attachment styles. It is a 4-item self-report measure that describes 4 prototypical attachment attitudes (secure, dismissing, preoccupied, and fearful), linked on a 7-point Likert scale. In this study, Cronbach α for RQ was 0.97.

#### Personality Domains

The Italian version [56] of the Personality Inventory for DSM-5—Brief Form—Adult (PID-5-BF [57]) was used to evaluate personality functioning. The PID-5-BF comprises 25 self-reported items designed to assess 5 maladaptive personality domains: negative affectivity, detachment, antagonism, disinhibition, and psychoticism, in accordance with the alternative *DSM-5* model for personality disorders [58]. Participants rate each item on a 4-point Likert scale, with higher scores indicating greater personality dysfunction. In this study, Cronbach α for PID-5-BF was 0.87. As for the Italian version of the RQ [54], the psychometric properties and validation processes of the translated version of the PID-5 are detailed in the original validation studies.

#### Fear of Intimacy With Helping Professions

Originally developed to assess the comfort of revealing intimate personal details to helping professions, the Fear of Intimacy With Helping Professionals Scale (FIS-HP [46]), is a modified version of the 35-item Fear of Intimacy Scale (FIS) [59]. The FIS-HP has 18 items grouped into 3 factors, each formed by 6 items, named fear of sharing, openness to intimate sharing, and information sharing. Although a validated Italian translation of this scale is not available, in this study, the original FIS-HP [42] was translated into Italian by 2 bilingual psychologists and researchers and then back-translated by an independent translator to verify the equivalence of the translated scale to the original one, in line with the process followed to obtain the Italian standardized version of the 35-item FIS [60]. In this study, Cronbach α for FIS-HP was 0.94.

### Statistical Analysis

#### Power

To determine sample size, we computed an a priori power analysis for logistic regression using G*Power (version 3.1.9.2; Heinrich-Heine-Universität Düsseldorf) [61] as described by Aysel et al [62], which resulted in a recommended sample size of n=683 (α=.05; β=.95; odds ratio [OR] 1.3, 95% CI 1.15-1.47; critical *z* score=1.64).

#### Data Analytics Strategy

Given the polytomous and ordinal nature of the dependent variable (ie, willingness to address mental health conditions only via CA-based interventions, assessed using 5 categories that may be ordered according to the level of magnitude), we performed an ordinal logistic regressive model using the polr function in the MASS package of R (R Core Team, 2025), following the procedure described by Liang and Zhan [63]. This modeling approach was selected because the POM is particularly well-suited for handling ordinal outcome variables, where the distance between response categories cannot be assumed to be equal. Unlike traditional methods such as ANOVA, which require continuous dependent variables and assume homogeneity of variance across groups, POM retains the ordinal structure of the outcome and models the cumulative probability of being in or above a particular category. This allows for a more accurate and interpretable analysis of ranked responses. Furthermore, POM enables the inclusion of multiple predictor variables—both continuous and categorical—and yields interpretable outputs in the form of ORs, which is particularly advantageous when assessing complex psychological and demographic predictors. Thus, the POM offers a more robust and statistically appropriate framework for evaluating the ordinal response structure inherent to participants’ preferences for CA-based therapy [64,65].

Probabilities of the ordinal response variable were transformed into log-odds via logit function, as shown in Equation 1.

Equation 1: Logistic transformation of cumulative probabilities.


(1) $$\text{logit}\left(P(Y\geq j)\right)=\ln[\left(P(Y\geq j)/1-P(Y\geq j)\right)]=\beta_{0j}+\beta_{1j}X$$

In logistic regression, log-odds indicate the likelihood of being at or below a certain category level relative to being above it. A positive logit value suggests a higher likelihood of being in or below a particular category, while a negative value indicates a higher likelihood of being above that category. To express odds in terms of predicted probabilities, which are often easier to interpret, we used the transformation formula:

Equation 2: Conversion from log-odds to predicted probabilities.


(2) $$P(Y\geq j)=\exp(\beta_{0j}+\beta_{1j}X)/[1+\exp(\beta_{0j}+\beta_{1j}X)]$$

Where P(Y≥j) represents the predicted probability of the dependent variable Y being in or below category level *j*, *e* is the base of the natural logarithm (≃2.718), β_0j_ and β_1j_ are, respectively, intercept and coefficient for the independent variable X. As gender is a dummy variable, we used the following formula:

Equation 3: Predicted probability for dummy variable (gender).


(3) $$P(Y\geq j)=\exp(\beta_{0j}+\beta_{1j}\times \text{Gender})/[1+\exp(\beta_{0j}+\beta_{1j}\times \text{Gender})]$$

We used the POM, considering the effects of independent variables (IVs) on the dependent variable as equal across all categories. For instance, the OR of each IV is the same for each cut-off – (1) P[Y=1] versus P[Y≥2], (2) P[Y≤2] versus P[Y≥3], (3) P[Y≤3] versus P[Y≥4], and (4) P[Y≤4] versus P[Y≥5]. POM is the most frequently used logistic regression model for analyzing ordinal response variables [64,65]. However, it leads to strong assumptions that, if violated, may lead to incorrect data interpretations [66]. Checking assumptions is crucial to ensuring the validity of statistical analyses and to justify the choice of a model above others [67]. For example, in their review of clinical psychology studies involving linear regression, Ernst and Albers [68] found that only 2% of cases were both transparent and correct in reporting assumption checking, and a further 6% were transparent but incorrect, leading to serious inferential problems [68].

To test the proportional odds assumption (or parallel line assumption), we conducted the Brant test via the Brant R package [69]. If the *P* value of the Brant test is lower than .05, the proportional odds assumption should be considered violated. When data fail to satisfy the proportional odds assumption, a solution is fitting a NPOM or the PPOM [70]. The main difference between NPOM and PPOM is that while the first allows each of the IVs to vary across each category of ordinal response variable, the second just allows to vary only IVs that have violated the assumption.

## Results

Out of 876 students, 736 students (84.01% respondents) aged 18 to 85 years completed the survey. As outliers increase data variability and decrease statistical power, 1 participant (male, age 85 y) was manually removed. The final sample consisted of 735 participants (191 males, 25.98%; 543 females, 73.88%; and 1 missing) with a mean age of 27.69 (SD 12.31) years. The single case with missing gender data was handled using multiple imputation, ensuring that no data were excluded from the analyses due to this missing value. Although the age range of the sample was broad (18‐85 y), the distribution was moderately right-skewed, with a median age of 24 (IQR 19‐31) years, reflecting the student population from which the sample was drawn. The majority (n=561, 76.36%) were single or in a nonserious relationship, and the remaining 414 (56.3%) were married or in a stable union. On average, participants had 13.84 (SD 3.50) years of education. A total of 76 individuals (10.30%) reported a previous mental health condition, such as anxiety or depression; 127 (17.3%) reported frequent substance use (≥3 times/week) without meeting the criteria for abuse or dependence.

Table 2 shows the POM model results.

The results of the Brant test show that the overall test fails, and the parallel line assumption is not satisfied (*χ*²_105_=187.8; *P*<.001). Thus, the results of POM should not be interpreted nor generalized, as some predictors might have nonuniform effects across different levels of the outcome variable, necessitating a more flexible modeling approach. In light of this, possible alternative models to explain the data were considered, and Tables3  4 report the results of such alternative models, respectively NPOM and PPOM.

After partially lifting the restriction, both PNOM and PPOM were models found to fit data correctly and may be useful to explain them. To identify the best solution for this purpose, we compared NPOM and PPOM by using residual deviance, which may be considered a measure of discrepancy of a generalized linear model from real observed data [71]. Thus, the lower the value, the smaller the discrepancy and the better the model can predict the value of the response variable. Residual deviance was computed by the VLMC R package [72], which allows for the optimization of the Akaike information criterion (AIC) or Bayesian information criterion (BIC) and overcomes problems related to residuals for polytomous response data by generating data through a Monte Carlo Chain. Results showed that PPOM had a better fit with respect to NPOM (r_D_=3530.472 vs 6244.009) and was therefore selected as the chosen model. More specifically, the choice of PPOM over the NPOM was justified by several considerations. First, the residual deviance for PPOM (3530.472) was significantly lower than that of NPOM (6244.009), indicating that PPOM provided a better fit to the observed data. While NPOM relaxed the proportional odds assumption entirely, allowing all predictors to vary across response levels, this approach risked overfitting and reduced parsimony, especially when predictors with no significant nonproportionality were unnecessarily freed. PPOM, on the other hand, selectively relaxed the proportional odds assumption only for predictors that violated it, as identified through the Brant test. This selective relaxation struck a balance between addressing assumption violations and maintaining interpretability, which was a key advantage over the more complex NPOM. By preserving the proportional odds assumption for variables that met it, PPOM simplified interpretation while capturing nuanced effects for predictors like relationship status and fear of sharing. The findings from PPOM offered unique insights. For instance, epistemic trust demonstrated a consistent effect across all response levels, underscoring its robust role as a predictor of preference for CA-based therapy. Fear of sharing, however, exhibited a nonuniform effect that intensified as willingness thresholds increased. Similarly, PPOM revealed a gradient effect for relationship status, where single individuals showed the strongest preference for CA-based therapy at lower thresholds, a pattern that diminished at higher thresholds. Traits like detachment and psychoticism also varied across levels, with detachment exerting a weaker influence at lower willingness thresholds and becoming more pronounced at higher thresholds. These level-specific effects, which were not as clearly identifiable in NPOM, underscored the nuanced contributions of PPOM. Therefore, the model provided a more precise and actionable understanding of the factors influencing CA-based therapy preferences, balancing statistical rigor with interpretability and offering practical insights for targeted interventions.

**Table 2.** Proportional odds model results.

Variables	OR^a^	SE	*z* value	*P* value
Intercept 1	1.400	0.853	0.395	.69
Intercept 2	0.852	0.851	−0.188	.85
Intercept 3	0.367	0.851	−1.180	.24
Intercept 4	0.155	0.853	−2.188	.03
Gender (male)	0.724	0.204	−1.585	.11
Age (years)	1.000	0.009	0.005	≥.99
Education (years)	1.082	0.025	3.170	.002
Single	3.717	0.389	3.378	<.001
Married or in a stable union	2.751	0.34	2.982	.003
Not in a serious relationship	1.572	0.747	0.605	.55
ETMCQ^b^				
Epistemic trust	1.745	0.434	4.017	<.001
Epistemic mistrust	0.727	0.52	1.398	.16
Epistemic credulity	0.677	0.675	1.002	.32
PID5−BF^c^				
Negative affectivity	1.014	0.056	0.253	.80
Detachment	0.95	0.065	14.615	.001
Antagonism	0.89	0.07	12.714	.001
Disinhibition	1.05	0.06	17.5	.002
Psychoticism	1.12	0.08	14.0	.003
RQ^d^				
Secure	1.040	0.054	0.731	.47
Fearful-avoidant	0.945	0.059	−0.955	.34
Preoccupied	0.923	0.065	−1.23	.22
Dismissing-avoidant	0.952	0.055	−0.896	.37
FIS−HP^e^				
Fear of sharing	1.086	0.025	3.24	.001
Openness to intimate sharing	1.015	0.025	0.591	.56
Sharing information	0.988	0.029	−0.406	.69

a OR: odds ratio.

b ETCMQ: Epistemic Trust, Credulity and Mistrust Questionnaire.

c PID5−BF: Personality Inventory for DSM-5—Brief Form—Adult.

d RQ: Relationship Questionnaire.

e FIS-HP: Fear of Intimacy with Helping Professionals Scale

**Table 3.** Results of the nonproportional odds model (NPOM).

Variables	logitlink(P[Y>=2])^a^	logitlink(P[Y>=3])	logitlink(P[Y>=4])	logitlink(P[Y>=5])
Intercept^b^	0.141	0.111	0.110	0.319
Gender (male)^b^	0.613	0.855	0.823	0.766
Age (years)^b^	0.978	0.996	0.993	1.004
Education (years)^b^	1.132	1.112	1.089	1.063
Single^b^	10.309	6.469	7.862	2.627
Married/stable union^b^	6.074	3.877	5.452	2.268
Not in a serious relationship^b^	2.662	3.068	2.199	1.465
ETMCQ^c^				
Epistemic trust^b^	1.117	1.114	1.111	1.070
Epistemic mistrust	0.85	0.88	0.87	0.89
Epistemic credulity^b^	1.154	1.137	1.099	1.076
PID5-BF^d^				
Negative affectivity^b^	1.110	1.083	1.100	0.988
Detachment^b^	0.877	0.902	0.910	1.011
Antagonism	0.141	0.111	0.110	0.319
Disinhibition	1.05	1.07	1.04	1.03
Psychoticism	1.10	1.08	1.06	1.07
RQ^e^				
Secure	0.948	1.037	1.089	1.028
Fearful-avoidant^b^	1.027	0.959	0.916	0.942
Preoccupied^b^	0.881	0.837	0.963	0.964
Dismissing-avoidant^b^	1.021	0.955	1.000	0.946
FIS-HP^f^				
Fear of sharing^b^	1.114	1.099	1.023	0.919
Openness to intimate sharing	1.241	1.182	1.124	0.969
Sharing information	0.969	0.968	1.002	0.965

a Logit link values represent log-odds coefficients from the ordinal logistic regression model, indicating the effect of each predictor on the likelihood of expressing greater willingness to engage in conversational agent–based therapy (measured on a 5-point ordinal scale). Positive coefficients indicate a higher probability of choosing more favorable response categories (e.g., “somewhat willing” or “extremely willing”), while negative coefficients reflect a tendency toward lower willingness.

b *P*<.001.

c ETCMQ: Epistemic Trust, Credulity and Mistrust Questionnaire.

d PID5−BF: Personality Inventory for DSM-5—Brief Form—Adult.

e RQ: Relationship Questionnaire.

f FIS-HP: Fear of Intimacy with Helping Professionals Scale

**Table 4.** Results of the partial proportional odds model (PPOM).

Variables	logitlink(P[Y>=2])	logitlink(P[Y>=3])	logitlink(P[Y>=4])	logitlink(P[Y>=5])
Intercept^a^	0.190	0.260	0.160	0.270
Gender (male)^a^	0.770	0.770	0.770	0.770
Age (years)^a^	1.000	1.000	1.000	1.000
Single^a^	18.03	8.020	9.830	2.420
Married or in a stable union^a^	7.710	4.540	6.100	2.170
Not in a serious relationship^a^	1.490	2.310	1.810	1.590
ETMCQ^b^				
Epistemic trust^a^	1.24	1.24	1.24	1.24
Epistemic mistrust^a^	0.93	0.93	0.93	0.93
Epistemic credulity^c^	1.09	1.09	1.09	1.09
PID-5BF^d^				
Negative affectivity^a^	1.010	1.010	1.010	1.010
Detachment^a^	0.970	0.970	0.970	0.970
Antagonism	0.950	0.950	0.950	0.950
Disinhibition	0.940	0.940	0.940	0.940
Psychoticism^a^	0.950	0.950	0.950	0.950
RQ^e^				
Secure	1.040	1.040	1.040	1.040
Fearful-avoidant^a^	0.940	0.940	0.940	0.940
Preoccupied^a^	0.950	0.950	0.950	0.950
Dismissing-avoidant^a^	0.960	0.960	0.960	0.960
FIS-HP^f^				
Fear of sharing^a^	1.03	1.03	1.02	1.01
Openness to intimate sharing	0.97	0.97	0.97	0.97
Sharing information	1.00	1.00	1.00	1.00

a *P*<.001.

b ETCMQ: Epistemic Trust, Credulity and Mistrust Questionnaire.

c *P*<.01.

d PID-5BF: Personality Inventory for DSM-5—Brief Form—Adult.

e RQ: Relationship Questionnaire.

f FIS-HP: Fear of Intimacy with Helping Professionals Scale

Overall, our data showed that epistemic trust emerged as a strong predictor of willingness to rely on CA-based therapy. Higher levels of epistemic trust significantly increased the likelihood of choosing AI-mediated therapy (OR 1.745, 95% CI 1.10‐1.41; *P*<.001). Fear of intimacy, particularly the subdomain fear of sharing, also demonstrated significant predictive power (OR 1.086, 95% CI 1.01‐1.06; *P*=.001). These findings suggest that individuals with higher epistemic trust and fear of interpersonal vulnerability are more inclined toward AI interventions. Attachment styles showed differential associations with preferences. Secure attachment was not a significant predictor, while avoidant attachment styles—particularly dismissing and fearful—were negatively associated with willingness to engage in CA-based therapy, although these effects were not statistically significant for fearful-avoidant style (*P*=.34), and should be interpreted cautiously. Personality traits, including negative affectivity, detachment, and psychoticism, were significant predictors of preference for CA-based therapy. For instance, detachment was associated with lower willingness (OR 0.95, 95% CI 0.94‐1.00; *P*<.001), while psychoticism predicted higher willingness (OR 1.12, 95% CI 1.04‐1.21; *P*=.003), suggesting that individuals with certain traits may prefer less emotionally intense interaction modalities. Demographic variables also contributed to the prediction model. Men were less likely than women to prefer CA-based therapy, although this effect was not statistically significant (*P*=.11). Education was positively associated with willingness to engage in CA therapy (OR 1.082, 95% CI 1.03‐1.14; *P*=.002), suggesting that more educated individuals may perceive AI as a viable therapeutic option. Although men were less likely than women to prefer CA-based therapy (OR 0.724, 95% CI 0.49‐1.08), this effect was not statistically significant (*P*=.11). Finally, relationship status showed robust associations: single participants were significantly more likely to prefer CA-based therapy (OR 3.717, 95% CI 1.73‐7.97; *P*<.001), as were those married or in stable unions (OR 2.751, 95% CI 1.41‐5.36; *P*=.003), when compared to those in less defined relationships. In sum, our analyses underscored the multifaceted role of epistemic trust, attachment, personality traits, and demographics in shaping individuals’ preferences for CA-based mental health interventions. These findings have implications for tailoring AI-based mental health services to individual needs and addressing potential barriers to their acceptance.

## Discussion

### Principal Findings

This study examined the psychological and demographic factors influencing individuals’ preferences for conversational agent (CA)-based therapy over traditional human-mediated interventions. Using robust statistical approaches, we identified significant predictors, including epistemic trust, attachment styles, personality traits, fear of intimacy, and demographic factors such as gender and education. More specifically, greater epistemic trust significantly increased individuals’ willingness to engage with CA-based mental health therapy, highlighting its central role as a precondition for trusting AI-mediated support. Higher levels of fear of intimacy, particularly fear of sharing personal information, also predicted a preference for CA-based support, suggesting that individuals who feel emotionally vulnerable or threatened by interpersonal closeness may gravitate toward less socially demanding therapeutic tools. Personality traits such as detachment and psychoticism were also associated with greater willingness to use AI-mediated therapy, reflecting a preference for emotionally distant or unconventional interaction styles. Attachment styles showed more nuanced effects: individuals with avoidant attachment (both dismissing and fearful) were less inclined to engage in traditional human-based therapy, indirectly favoring CA-based interventions. Demographic predictors such as being single and having a higher level of education further contributed to increased willingness to use CA-based mental health support. These findings align with broader trends in the literature. Recent studies demonstrate that user trust in AI mental health tools is influenced not only by personal traits but also by perceptions of safety, anonymity, and emotional support [3] For example, Abd-Alrazaq et al [71] found that emotional comfort and perceived nonjudgmentality foster trust in CAs - mirroring our finding that epistemic trust and fear of intimacy shape AI preferences. Similarly, a validated model by Lee et al [73] emphasizes the roles of affect, social norms, and habitual use in shaping engagement with well-being chatbots, supporting the notion that behavioral and contextual factors interact with internal dispositions, such as attachment and personality. Furthermore, a recent expert on AI-driven CAs for mental health support [12] raised concerns about user overreliance and the limited emotional depth of chatbot responses, highlighting ethical risks, particularly for individuals whose psychological profiles may predispose them to overuse AI to avoid vulnerability. A scoping review by Rahsepar Meadi et al [74] further underscored the need for transparency, informed consent, and safeguarding in AI therapy design, especially for emotionally avoidant users. Finally, a study analyzing users’ reviews of CA-based apps for mental health [75] found that users are more likely to disclose sensitive information to chatbots due to their perceived anonymity, reinforcing our interpretation that individuals with a fear of intimacy or high detachment may favor CAs as emotionally safer alternatives. Together, these converging lines of evidence reinforce our conclusion that psychological vulnerabilities and individual differences significantly influence engagement with AI-based mental health interventions. This underscores the importance of tailoring such tools to users’ psychological needs while also addressing ethical, emotional, and relational implications of replacing human intimacy with machine-mediated interaction in mental health care [74]. The following sections delve deeper into the specific roles of epistemic trust, attachment styles, personality traits, and fear of intimacy, providing a more granular analysis of how each contributes to shaping individual engagement with AI-mediated therapy.

### Epistemic Trust and the Role of AI

The results underscored the centrality of epistemic trust in shaping individuals’ willingness to engage with CA-based therapy. Higher epistemic trust significantly predicted a greater preference for CAs (OR 1.745; 95% CI 1.31–2.32; *P*<.001), affirming its role as a key determinant of trust in AI-mediated interventions. This finding aligns with previous work [68], which emphasized the importance of trust in fostering acceptance of AI technologies. Our results suggest that individuals who perceive AI as a reliable source of information and support are more inclined to rely on it for mental health therapy, echoing the epistemic relationship outlined by Alvarado [22], who highlighted AI’s capacity to enhance human knowledge in epistemic contexts. These findings also resonate with the conceptualization of epistemic trust as a product of early social learning [22,69], which influences how individuals assess the reliability of new sources of knowledge, including AI. This study contributes to the growing evidence that AI systems, when perceived as epistemically trustworthy, can be viable alternatives to human therapists. However, it also raises ethical considerations about overreliance on AI and the potential for reduced critical thinking, as highlighted in recent debates on AI’s role in health care [70].

### Attachment Styles and Fear of Intimacy

Attachment styles, particularly avoidant patterns, emerged as significant predictors of CA-based therapy preference. Individuals with dismissing and fearful attachment styles were less likely to engage with traditional human therapists, likely due to discomfort with emotional intimacy and vulnerability. These findings align with Bartholomew and Horowitz’s [43] model of attachment, which posits that dismissing individuals prioritize independence and avoid reliance on others, while fearful individuals desire connection but struggle with trust and fear of rejection. The preference for CAs may reflect a less threatening avenue for emotional expression, consistent with the argument that digital platforms offer “safe spaces” for intimacy without the pressures of face-to-face interaction [75]. Fear of intimacy, particularly fear of sharing, also significantly influenced preferences for CA-based therapy (OR 1.09, 95% CI 1.03–1.14; *P*=.001). This supports previous findings that individuals who struggle with interpersonal vulnerability may gravitate toward less invasive therapeutic options. Ingersoll et al [42] similarly found that fear of intimacy negatively impacts the therapeutic alliance, potentially hindering the effectiveness of traditional therapy. These dynamics underscore the need for CA-based therapies to account for such fears while ensuring they provide adequate emotional support and empathy. Interestingly, while dismissing and fearful-avoidant attachment styles were negatively associated with willingness to engage in CA-based therapy, consistent with their underlying discomfort with closeness or trust, secure attachment did not significantly predict CA preference. This may reflect the adaptive flexibility of securely attached individuals, who are typically comfortable with both intimacy and autonomy [76,77]. As such, they may evaluate AI and human-delivered interventions based more on perceived usability or relevance than on relational needs or avoidance patterns. In this context, their openness to various forms of support may lead to a neutral or balanced preference, rather than a strong inclination toward or against CA-based therapy. Future studies might explore this by directly comparing secure individuals’ preferences across therapeutic formats under varying emotional and contextual demands.

### Personality Traits and Preferences for AI Therapy

Personality traits, particularly negative affectivity, detachment, and psychoticism, were significant predictors of willingness to engage with CA-based therapy. These traits reflect tendencies toward emotional dysregulation, social withdrawal, and unconventional thinking, which may drive preferences for less interpersonal forms of therapy. The findings align with Marengo et al [30] who noted that personality traits significantly influence how individuals interact with digital technologies, including AI. The association between detachment and CA preference (OR=0.95, *P*<.001) is particularly notable. Detachment often involves avoidance of emotional closeness, making traditional therapy challenging. CAs, perceived as emotionally neutral, may offer a more comfortable alternative. This supports prior research by Matthews et al [27] who remarked that personality traits affect individuals’ comfort with AI, particularly in emotionally charged contexts like therapy. Similarly, the significant role of psychoticism (OR 1.12, 95% CI 1.04–1.21; *P*=.003) may reflect the appeal of AI-mediated therapy for individuals with unconventional cognitive styles who might find human interactions overwhelming or judgmental.

### Demographic Influences on CA-Based Therapy Preferences

Demographic factors, including gender, education, and relationship status, also played a role in shaping preferences for CA-based therapy. While gender differences were not statistically significant, the trend suggested that men were slightly more inclined toward AI therapy than women. This aligns with previous findings suggesting that men are generally more open to adopting new technologies [28]. Education level was positively associated with willingness to engage in CA therapy, suggesting that individuals with higher education levels may perceive AI as a credible and innovative tool for mental health support. These findings corroborate Glikson and Woolley’s [68] assertion that technological literacy influences the acceptance of AI in various domains. Relationship status also emerged as a significant predictor, with single individuals showing a markedly higher preference for CA-based therapy compared to those in stable unions (OR 3.72, 95% CI 2.37–5.82; *P*<.001). This may reflect the increased reliance on digital solutions for emotional support among individuals with less access to intimate interpersonal relationships, consistent with findings by Loveys et al [2] on the role of digital technologies in mitigating loneliness.

### Implications for Practice and Policy

The findings have several practical and ethical implications. First, they highlight the need for CA-based therapies to be designed with sensitivity to individual differences, particularly attachment styles and personality traits. For instance, individuals with high detachment may benefit from interventions that gradually build emotional engagement, while those with high epistemic trust may prioritize transparency and reliability in AI responses. Based on these findings, we recommend that developers incorporate adaptive interaction styles into CA systems, such as pacing strategies, tailored disclosure prompts, and emotionally neutral yet supportive communication styles, to accommodate users who struggle with emotional closeness or fear of judgment. Second, our results underscore the importance of addressing the potential risks associated with CA-based therapy. While these technologies offer valuable alternatives, particularly for individuals who struggle with traditional therapy, they also carry risks of overreliance and reduced critical thinking [74]. As discussed by Abd-Alrazaq et al [3], ensuring that CA-based therapies are used as adjuncts rather than replacements for human interaction is critical to maintaining a balanced approach to mental health care. Third, the role of demographic factors suggests that targeted outreach and education efforts may be necessary to increase the accessibility and acceptance of CA-based therapies across diverse populations. For example, addressing technological skepticism among older adults and less educated individuals could help bridge gaps in mental health support. Furthermore, we suggest that mental health practitioners consider screening tools for psychological traits such as epistemic trust, attachment insecurity, and fear of sharing, to help determine whether CA-based interventions are suitable entry points or supportive complements to traditional therapy. CAs could then be integrated into care in a way that accounts for users’ emotional readiness and interpersonal vulnerabilities. Developers might also consider implementing real-time monitoring features within CA systems to detect signs of excessive dependency or emotional withdrawal, prompting referral to human practitioners when necessary. Finally, the findings raise important ethical questions about the integration of AI in mental health care. As highlighted in a recent review [78], the design and implementation of AI systems must prioritize patient safety, privacy, and informed consent. These concerns are echoed in emerging frameworks for responsible AI integration in mental health research, which emphasize transparency, stakeholder inclusion, and regulatory oversight as essential components of ethical deployment [79]. These considerations are particularly important given the reliance on self-reported data and the potential for biases in AI algorithms. Taken together, our findings provide a foundation for designing CA systems that are both ethically responsible and psychologically attuned to the individual needs of users.

### Strengths, Limitations, and Future Directions

This study offered a novel contribution to the emerging field of digital mental health by focusing not only on the technological acceptability of CAs, but also on the psychological determinants that underpin individual preferences for AI-mediated therapy. While previous research has highlighted user satisfaction, perceived usefulness, or the technical capabilities of CAs, relatively little attention has been paid to why certain individuals are more psychologically predisposed to prefer AI over human interaction in therapeutic contexts. By integrating constructs such as epistemic trust, fear of intimacy, attachment style, and maladaptive personality traits, this study provides a more comprehensive and person-centered model of CA engagement. This approach fills a critical gap in the literature and offers clinically relevant insights for tailoring CA-based interventions to the psychological needs and vulnerabilities of different user profiles. Nonetheless, while providing valuable insights, this study is not without limitations. The sample was drawn from a specific population (university students), which may limit the generalizability of the findings. More specifically, the participants were all enrolled in a psychology degree program, which likely reflects a cohort that is highly educated, psychologically literate, and potentially more open to digital therapeutic tools than the general population. This homogeneity may have biased responses toward higher levels of epistemic trust and engagement with AI, which could inflate associations between psychological variables and preference for CA-based therapy. As noted in broader critiques of online survey methodology (eg, Andrade [80]), such sampling bias is common and warrants careful consideration when interpreting results. Future studies should replicate this work using more diverse and representative populations to validate and extend the findings. In addition, the reliance on self-reported measures introduces the possibility of response bias, particularly in relation to sensitive topics such as mental health history or substance use. While all participants in this study were aware of what CAs and CA-based therapy entail, due to their academic exposure to topics including the definition, clinical implications, and ethical considerations of AI-mediated therapy, this context should not be conflated with broader public understanding. Although we did not assess participants’ personal use of AI tools for psychological support, this was a deliberate methodological choice, as the study aimed to investigate individuals’ willingness to engage with CA-based therapy, not their actual behavioral engagement. Nonetheless, individual differences in previous exposure to AI may have shaped interpretations, and this represents an important avenue for future research. Finally, we acknowledge that although the study was grounded in established psychological theory, its cross-sectional design limits the ability to draw causal conclusions. While we refer to associations between constructs such as epistemic trust, attachment style, and CA preference, these relationships should be interpreted as correlational. Longitudinal or experimental designs are needed to determine causal pathways or mediating processes underlying these associations. Looking ahead, while this study focused on individual psychological predictors, future work could explore the interaction between user characteristics and system-specific features, such as CA interactivity, tone, or anthropomorphism. Investigating how these elements jointly influence engagement and outcomes could offer richer insights into optimizing AI-supported mental health interventions across diverse user groups. This priority also aligns with recent reviews (eg, Beg et al [81]) calling for research that connects user characteristics with CA functionalities, including interface style, responsiveness, and therapeutic framing.

### Conclusion

This study contributes to the growing literature on AI-mediated mental health interventions by identifying key psychological and demographic predictors of preferences for CA-based therapy. The findings emphasize the importance of epistemic trust, attachment styles, personality traits, and demographic factors in shaping attitudes toward AI in mental health care. While these technologies offer promising alternatives to traditional therapy, their integration must be approached with caution to ensure ethical and effective mental health support. Future research should continue to explore the dynamic interactions between individual differences and technological features, paving the way for personalized and equitable mental health interventions.

## Abbreviations

AI: artificial intelligence
CA: conversational agent
CHERRIES: Checklist for Reporting Results of Internet E-Surveys
ETCMQ: Epistemic Trust, Credulity and Mistrust Questionnaire
FIS: Fear of Intimacy Scale
FIS-HP: Fear of Intimacy with Helping Professionals Scale
IV: independent variable
NPOM: nonproportional odds model
OR: odds ratio
PID-5-BF: Personality Inventory for DSM-5—Brief Form—Adult
PIS: Participation Information Sheet
POM: proportional odds model
PPOM: partial proportional odds model
RQ: Relationship Questionnaire
